# Multifocal Head and Neck Paragangliomas: A Case Report of Bilateral Carotid Body Tumors With DOTANOC-Avid Thyroid Lesion

**DOI:** 10.7759/cureus.104223

**Published:** 2026-02-25

**Authors:** S Rohith, Priya Mani, Venkata Sai, Rajeswaran Rangasami, Subalakshmi Balasubramanian

**Affiliations:** 1 Department of Radiology, Sri Ramachandra Institute of Higher Education and Research, Chennai, IND; 2 Department of Pathology, Sri Ramachandra Institute of Higher Education and Research, Chennai, IND

**Keywords:** carotid body tumors, dotanoc-avid lesion, neuroendocrine tumors, paragangliomas, shamblin classification

## Abstract

Carotid body tumors (CBTs) are rare neuroendocrine neoplasms arising from paraganglionic tissue at the carotid bifurcation. Bilateral and multifocal involvement is uncommon and may indicate an underlying hereditary predisposition. We report a case of a 32-year-old man who presented with bilateral neck swellings and was diagnosed with bilateral CBTs and a DOTANOC-avid thyroid nodule. Multimodality imaging, including CT angiography and Ga-68 DOTANOC PET/CT, demonstrated hypervascular lesions with characteristic carotid artery splaying and variable degrees of vascular encasement. Imaging played a crucial role in lesion characterization, assessment of tumor extent, detection of multifocal disease, and surgical planning. Histopathological evaluation confirmed the diagnosis of paraganglioma. This case highlights the importance of comprehensive imaging evaluation in patients with suspected paragangliomas and emphasizes the need to consider genetic evaluation and long-term surveillance in multifocal presentations.

## Introduction

Carotid bodies are specialized peripheral chemoreceptors located in the adventitial layer of the carotid arteries, usually at the bifurcation. They are the largest paraganglia in the head and neck region. Until the adrenal glands mature, carotid bodies are a major source of catecholamines during fetal life.

Paragangliomas are rare neuroendocrine tumors that develop from paraganglionic cells, with carotid body tumors (CBTs) being the most prevalent at head and neck sites. These tumors represent a small percentage of neoplasms in the head and neck region, accounting for approximately 0.5% of cases [1]. Although CBTs are neuroendocrine neoplasms, they rarely produce catecholamines, and most are benign. While bilateral or multifocal disease raises the possibility of hereditary syndromes such as SDHx mutations and familial PGL gene locus (situated at chromosome 11q23), the majority of CBTs are unilateral and sporadic [2,3].

Paragangliomas are hypervascular tumors, and complete surgical excision is the gold standard treatment [4]. Advanced cross-sectional and functional imaging aids in detecting tumor multiplicity, delineating vascular relationships, and guiding multidisciplinary decision-making. Further, in head and neck paragangliomas, given the uncommon nature of such presentations and the possible implications for surgical approach, perioperative risk stratification, and consideration of underlying genetic associations, radiological investigation can play a major role.

This case highlights the diagnostic and management challenges posed by CBTs with suspected multifocal involvement, where accurate preoperative imaging characterization played a crucial role in optimal surgical planning.

## Case presentation

A 32-year-old man presented in October 2025 with gradually progressive bilateral neck swellings for three months. There were no symptoms of catecholamine excess. There was no significant family history. Clinical examination showed firm, pulsatile swellings in the upper cervical region. The patient was advised to undergo CT angiography for further evaluation.​​​​​​

Imaging findings

**Figure 1:**
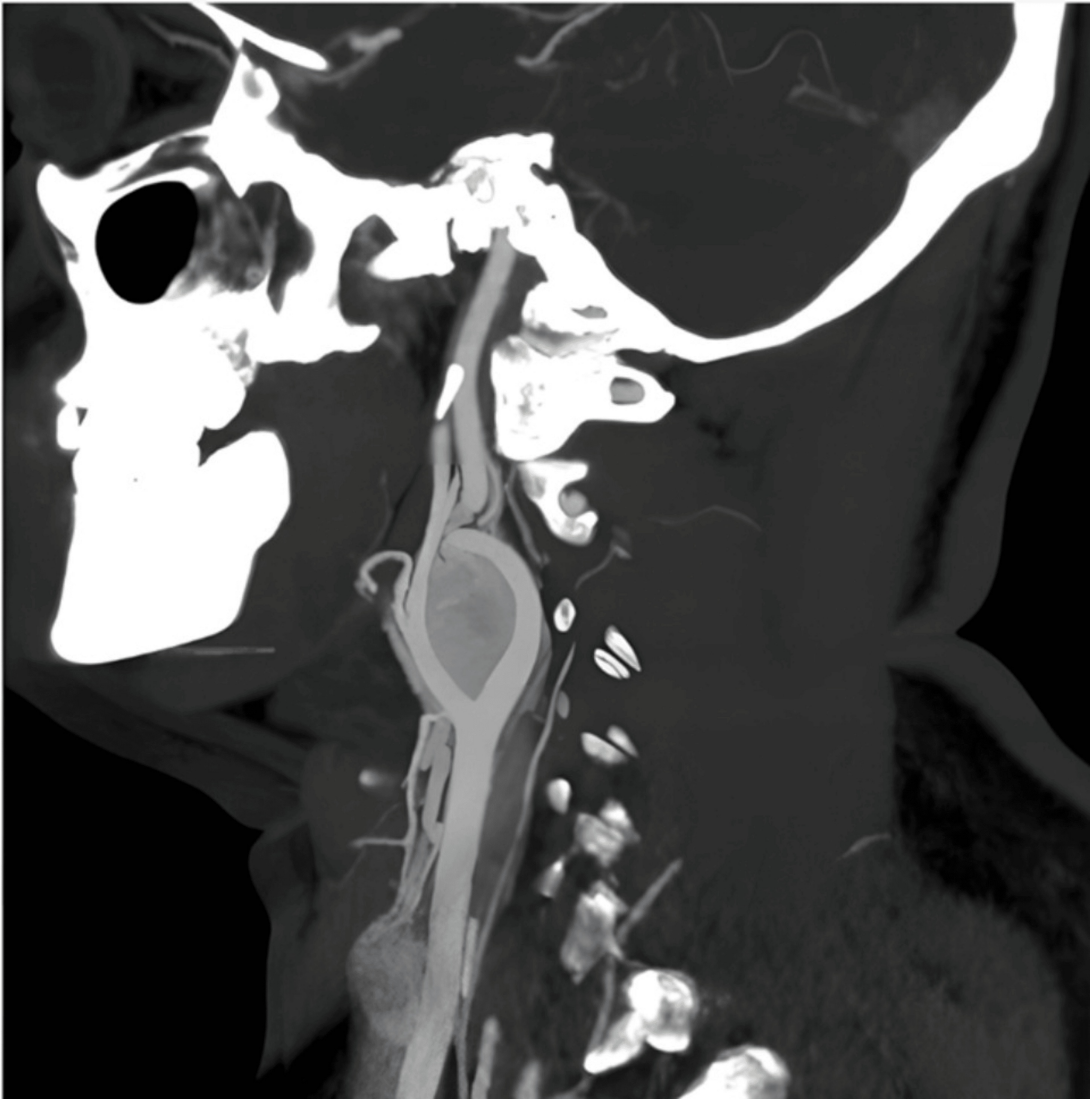
Right carotid space: a well-defined heterogeneously enhancing soft tissue density lesion with few nonenhancing areas within (representing necrosis) at the level of upper border of C3 to lower border of C4 vertebra causing splaying of the right ECA and ICA ICA: internal carotid artery; ECA: external carotid artery

CT angiography revealed bilateral neck masses at the carotid bifurcation. A well-defined heterogeneously enhancing soft tissue density lesion with few nonenhancing areas within (representing necrosis) was noted in the right carotid space, measuring ~ 2.5 x 2.0 x 2.5 cm (anteroposterior (AP) × transverse (TR) × craniocaudal (CC)) at the level of the upper border of the C3 to the lower border of the C4 vertebra. It was noted to cause splaying of the right external and internal carotid arteries. The angle of contact with the internal carotid artery (ICA) was 180°-270° (Shamblin II). The angle of contact with the external carotid artery (ECA) was 180°-270° (Figure 1).

**Figure 2:**
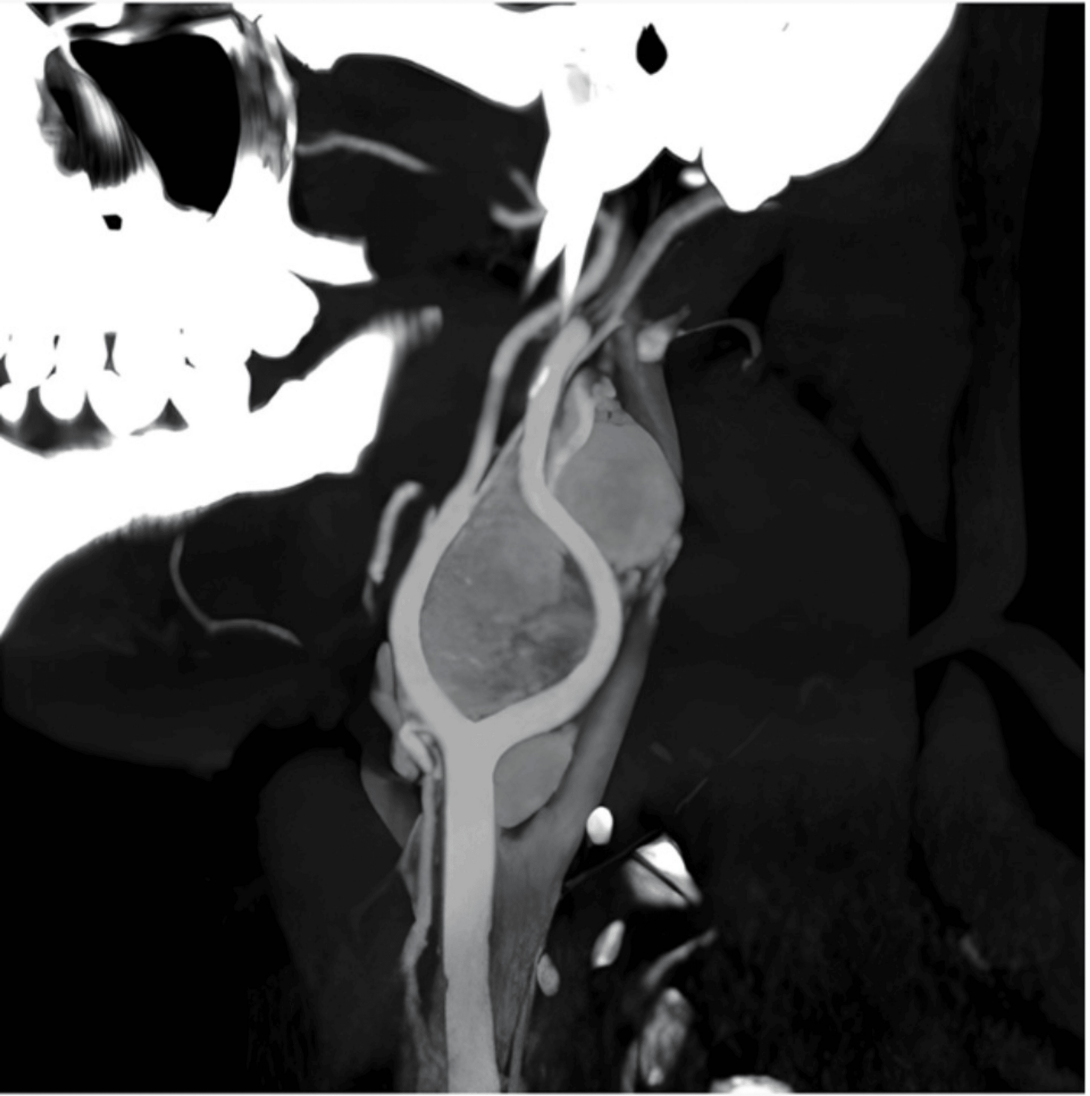
Left carotid space: a well-defined heterogeneously enhancing soft tissue density lesion with few nonenhancing areas within (representing necrosis), causing splaying of left ICA and ECA with multiple arterial feeders within the lesion supplied by its branches. Another well-defined heterogeneously enhancing soft tissue density lesion with few nonenhancing areas within (representing necrosis) noted lateral to the left ECA from the superior border of C2 to inferior border of C2 vertebra ICA: internal carotid artery; ECA: external carotid artery

A well-defined heterogeneously enhancing soft tissue density lesion with few nonenhancing areas (representing necrosis) was noted in the left carotid space, measuring ~ 2.8 x 4.1 x 5.2 cm (AP x TR x CC). It was noted to cause splaying of the left external and internal carotid arteries. The angle of contact with ICA was 270°-360° (Shamblin III). The angle of contact with the ECA was 360°, and the angle of encasement with the distal CCA was 180°-270°. Multiple arterial feeders were noted within the lesion supplied by ICA and ECA branches (Figure 2).

Another well-defined heterogeneously enhancing soft tissue density lesion with few nonenhancing areas (representing necrosis) measuring ~ 1.7 x 1.6 x 2.0 cm was noted lateral to the left ECA from the superior border of the C2 to the inferior border of the C2 vertebra (Figure 2).

**Figure 3:**
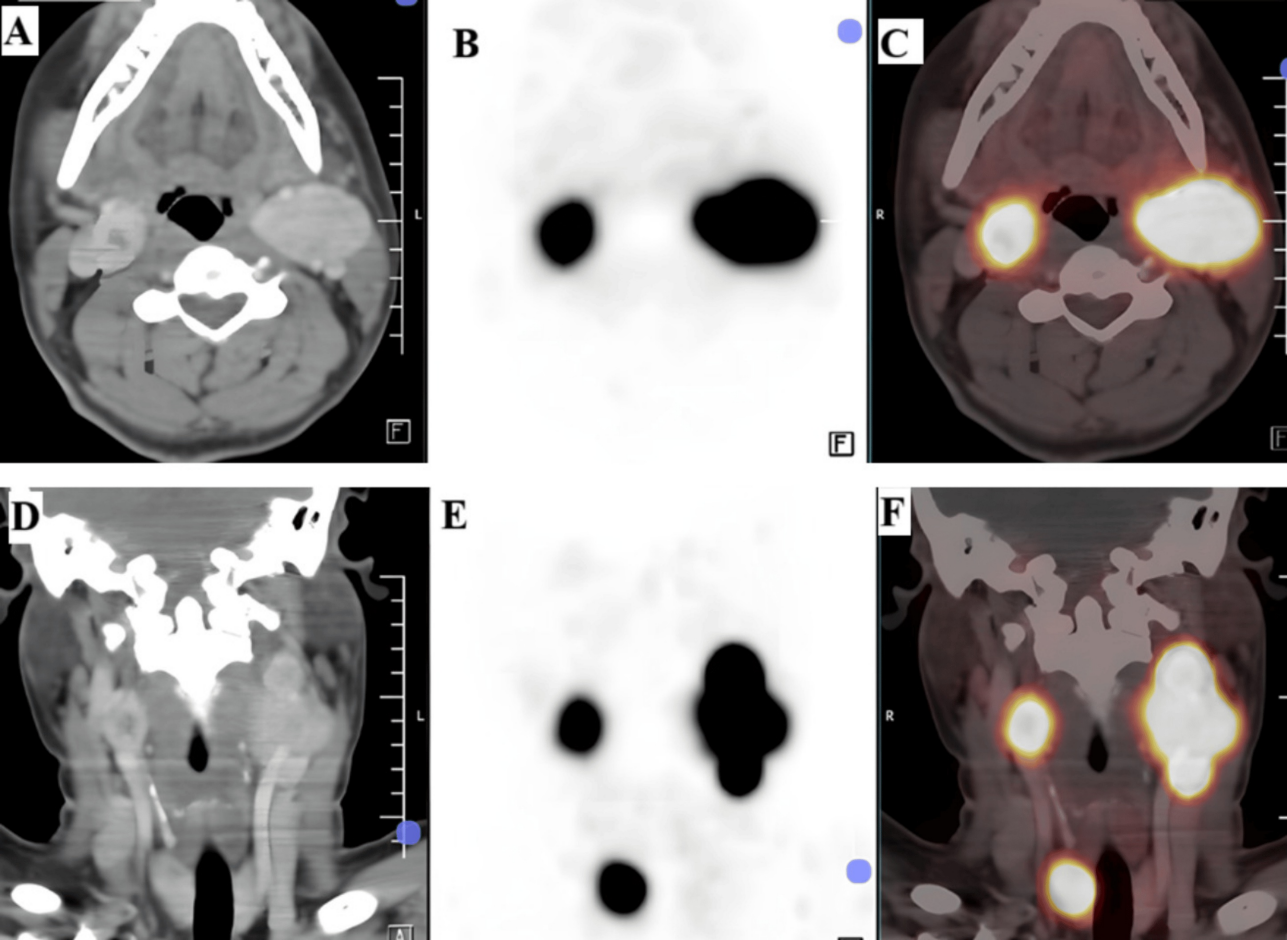
Ga-68-DOTANOC PET/CT demonstrating intense tracer avidity in bilateral carotid space lesions and additional foci (A) Axial contrast-enhanced CT image at the level of the carotid bifurcation demonstrating well-defined enhancing soft tissue lesions in the bilateral carotid spaces, splaying the internal and external carotid arteries. (B) Axial PET image showing increased radiotracer uptake corresponding to the carotid space lesions. (C) Axial fused PET/CT image demonstrating intense DOTANOC avidity in the bilateral carotid space lesions, with higher uptake in the left-sided lesion (SUVmax 54.3) compared to the right-sided lesion (SUVmax 28.36). (D) Coronal contrast-enhanced CT image demonstrating bilateral carotid space masses and an additional lesion lateral to the left external carotid artery. (E) Coronal PET image showing tracer-avid lesions corresponding to the carotid space masses and an additional inferior focus. (F) Coronal fused PET/CT image demonstrating intense DOTANOC uptake in the bilateral carotid space lesions, markedly increased avidity in the lesion lateral to the left external carotid artery (SUVmax 149.2), suggestive of high somatostatin receptor density, and additional DOTANOC avid focus in the right jugular bulb region (SUVmax 4.0) SUV: standardized uptake value

On DOTANOC scan, the lesions showed avidity; the right carotid space lesion had a standardized uptake value (SUV) max of 28.36, and the left carotid space lesion, causing splaying of ICA and ECA, had an SUVmax of 54.3. The lesion lateral to the left ECA had an SUVmax of 149.2. A DOTANOC avid (SUVmax 4.0) focus was noted in the region of the right jugular bulb (Figure 3).

**Figure 4:**
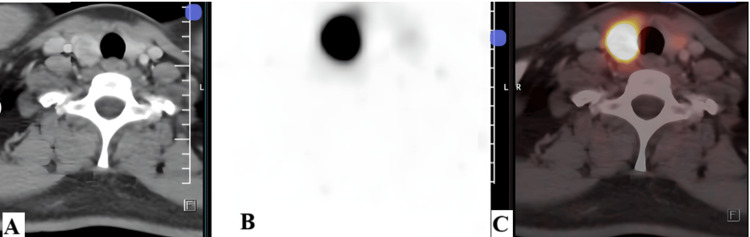
Ga-68-DOTANOC PET/CT demonstrating DOTANOC-avid thyroid nodule (A) Axial contrast-enhanced CT image at the level of the thyroid gland demonstrating a well-defined, arterially heterogeneously enhancing nodule in the right lobe of the thyroid. The left thyroid lobe appears grossly unremarkable. (B) Axial PET image showing focal increased radiotracer uptake corresponding to the right thyroid lobe lesion. (C) Axial fused PET/CT image demonstrating intense DOTANOC avidity within the right thyroid nodule (SUVmax 67.8), suggestive of significant somatostatin receptor expression SUV: standardized uptake value

A well-defined, arterially heterogeneously enhancing DOTANOC-avid (SUVmax 67.8) nodule of size ~1.4 x 2.0 x 1.7 cm was noted in the right lobe of the thyroid. The left lobe appeared grossly normal (Figure 4).

Treatment and histopathology

The patient was advised of the intervention and underwent surgical excision under general anesthesia with strict aseptic precautions. Following standard skin preparation and draping, a cervical incision was made along the anterior border of the sternocleidomastoid muscle and deepened through the platysma. The internal jugular vein was identified, looped, and secured, following which the facial vein was isolated and ligated. The common, internal, and external carotid arteries were subsequently identified, looped, and controlled. The primary lesion was carefully dissected and separated from the common, internal, and external carotid arteries and excised in toto.

In addition, a second mass was identified along the course of the vagus nerve. Proximal and distal control of the vagus nerve was achieved, and the lesion was completely excised while preserving nerve continuity. Adequate hemostasis was secured, a suction drain was placed, and the wound was closed in anatomical layers.

Following handover from the vascular surgery team, the incision was extended along a natural horizontal skin crease on the right side in the form of a Kocher’s incision. The skin and subcutaneous tissues were dissected, and subplatysmal flaps were elevated superiorly up to the level of the thyroid notch and inferiorly to the sternal notch. The linea alba was identified and retracted from the midline. The right sternothyroid muscle was retracted laterally and transected superiorly to improve exposure.

The superior pedicle of the thyroid gland was identified, and the superior thyroid artery was isolated, ligated, and divided. The right thyroid lobe was then mobilized from its lateral attachments. The inferior thyroid artery was identified, ligated, and divided. The right recurrent laryngeal nerve was carefully identified and preserved throughout the procedure. The right thyroid lobe was dissected free from Berry’s ligament and excised in toto.

**Figure 5:**
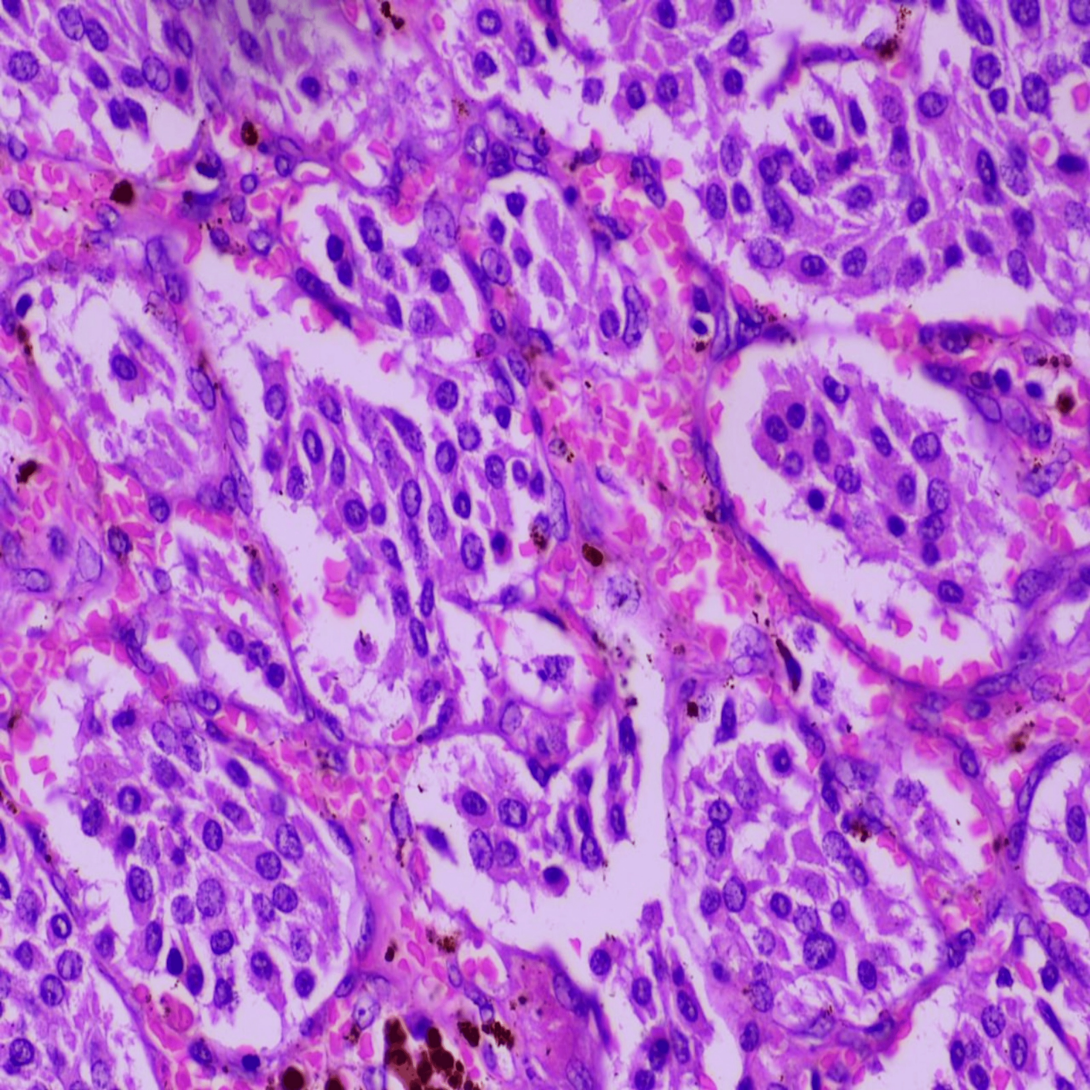
Highly cellular neoplasm composed of nests and lobules of uniform tumor cells arranged in a characteristic zellballen pattern, separated by delicate fibrovascular septae

**Figure 6:**
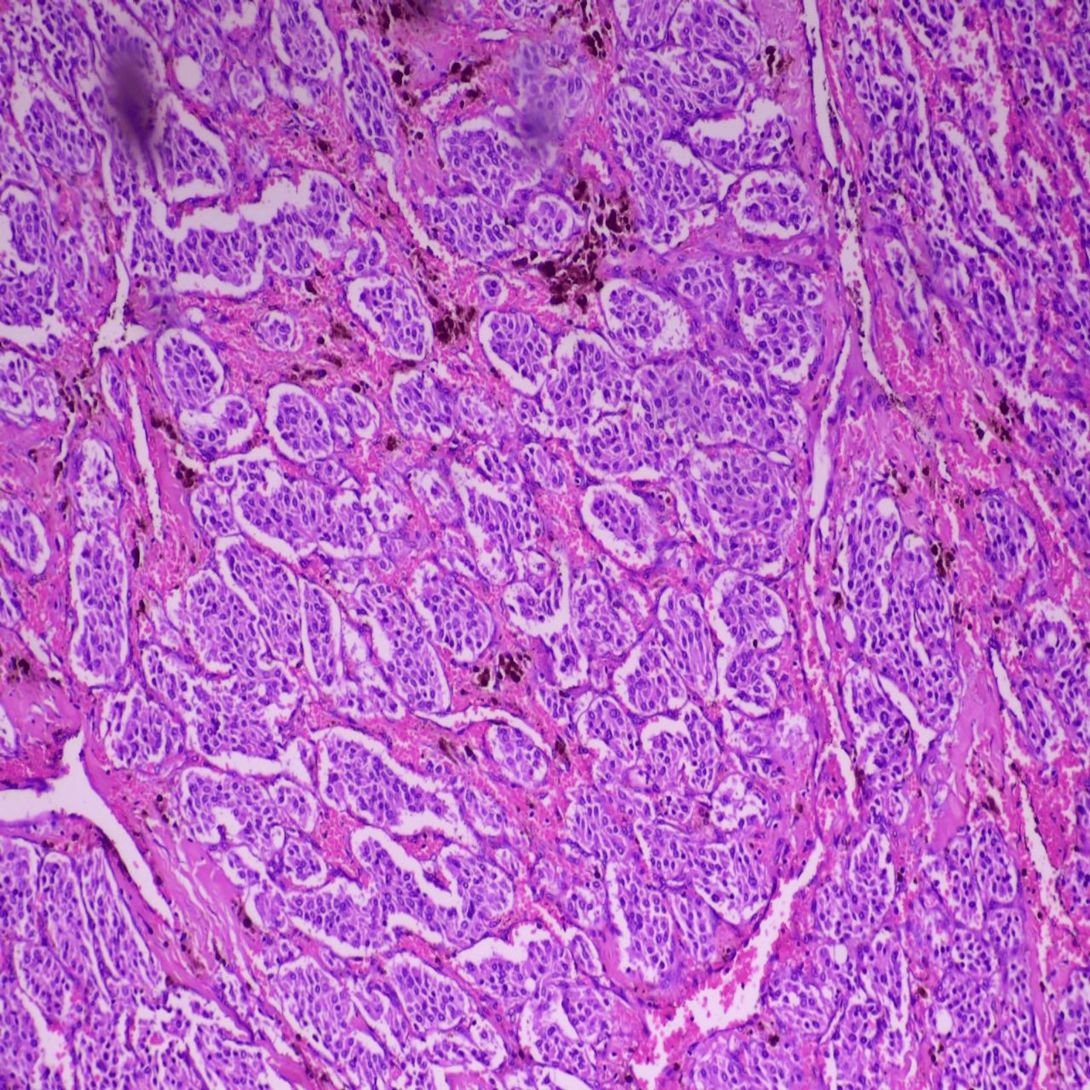
The tumor cells were round to polygonal with moderate eosinophilic granular cytoplasm and centrally placed round to oval nuclei exhibiting finely stippled (salt-and-pepper) chromatin. Nuclear pleomorphism was minimal, and mitotic figures were inconspicuous. The intervening stroma showed prominent capillary-sized blood vessels with focal areas of vascular congestion and hemorrhage. No evidence of tumor necrosis, significant mitotic activity, or features suggestive of malignancy was identified in the examined sections

The specimens were sent for histopathological examination (Figures 5, 6), and the morphological features were consistent with paraganglioma, correlating with the clinical diagnosis of a CBT.

## Discussion

In the published literature, CBTs have historically been characterized as hypervascular paragangliomas around the carotid bifurcation, classically noted for avid enhancement and typical splaying of the internal and external carotid arteries on cross-sectional studies. When patients present with bilateral CBTs, a detailed evaluation of both carotid bifurcations and other paragangliomas should occur due to this relatively uncommon manifestation of CBTs, as noted in a scholarly article by Patil et al. that explored this topic in detail [2]. Bilateral and multifocal paragangliomas are rare and strongly suggest a hereditary etiology. SDHx mutations, specifically SDHD and SDHB, are linked to 30%-50% of head and neck paragangliomas. Thyroid neuroendocrine lesions and multifocal tumors have been reported in SDHB-associated syndromes.

Based on a review of scholarly work by Hoang et al., CT angiography/magnetic resonance angiography are important for characterizing tumor extent and relationships with other vascular structures, which play an integral role in operative difficulty and risk in surgical repairs [5,6]. The Shamblin classification aids in predicting surgical complexity, with Shamblin II and III tumors presenting greater operative risks because of vessel encasement. Functional imaging, like DOTANOC PET/CT, is essential for preoperative mapping and is better at identifying multifocal disease.

Several case reports, such as that by Alqhtani et al., focus on CBT presentation and treatment planning; they also underscore that CBTs may occasionally present bilaterally and that clinicians should maintain suspicion for multifocality when clinical or imaging cues suggest it. In comparison, our case is notable for the combination of bilateral CBTs with additional DOTANOC-avid paraganglionic foci and a DOTANOC-avid thyroid nodule, where functional imaging (Ga-68 DOTANOC PET/CT) complemented CT angiography by improving whole-body lesion detection and preoperative mapping, thereby strengthening confidence in disease multiplicity and extent. In cases associated with thyroid DOTANOC-avid lesions, serum calcitonin and a potential fine needle aspiration cytology or biopsy should be performed to rule out medullary thyroid carcinoma, another neuroendocrine tumor.

From a management perspective, Piazza et al. outline practical strategies for bilateral CBTs, including staged approaches and tailoring the sequence of surgery to reduce cranial nerve and vascular complications in more complex tumors [7]. Resection of bilateral CBTs can lead to complications such as baroreflex failure syndrome, causing constant hypertension during the first 24-72 hours after surgery, followed by hypotension, headache, emotional instability, and palpitations. Management involves staged surgical resection, prioritizing the less complex lesion. For very vascular and large CBTs, embolization prior to surgery has demonstrated safer resection by decreasing vascularity and dimensions of the tumor [8]. Alternative treatment options such as radiotherapy and chemotherapy can be considered when a surgical approach is not feasible, but they are less effective. Additionally, genetic evaluation is essential for multifocal disease and surveillance planning.

Taken together, compared with predominantly single-lesion case descriptions, our report adds value by illustrating how combined anatomic and somatostatin-receptor-based functional imaging can clarify multifocal disease burden and support surgical planning and long-term surveillance considerations in a young patient with a complex distribution of lesions.

## Conclusions

This case encases a spectrum of multifocal head and neck paragangliomas in a young man, including a jugular bulb lesion, a DOTANOC-avid thyroid nodule, an accessory left paraganglioma, and bilateral CBTs (Shamblin II and III). For precise diagnosis, surgical planning, and the detection of additional lesions, comprehensive multimodality imaging is essential. Genetic testing should be strongly considered in such multifocal presentations.
